# Chronic captivity stress in wild animals is highly species-specific

**DOI:** 10.1093/conphys/coz093

**Published:** 2019-12-04

**Authors:** Clare Parker Fischer, L Michael Romero

**Affiliations:** Department of Biology, 200 College Ave. Tufts University, Medford, MA 02155 USA

**Keywords:** stress, captivity, glucocorticoids, reproduction, immune

## Abstract

Wild animals are brought into captivity for many reasons—conservation, research, agriculture and the exotic pet trade. While the physical needs of animals are met in captivity, the conditions of confinement and exposure to humans can result in physiological stress. The stress response consists of the suite of hormonal and physiological reactions to help an animal survive potentially harmful stimuli. The adrenomedullary response results in increased heart rate and muscle tone (among other effects); elevated glucocorticoid (GC) hormones help to direct resources towards immediate survival. While these responses are adaptive, overexposure to stress can cause physiological problems, such as weight loss, changes to the immune system and decreased reproductive capacity. Many people who work with wild animals in captivity assume that they will eventually adjust to their new circumstances. However, captivity may have long-term or permanent impacts on physiology if the stress response is chronically activated. We reviewed the literature on the effects of introduction to captivity in wild-caught individuals on the physiological systems impacted by stress, particularly weight changes, GC regulation, adrenomedullary regulation and the immune and reproductive systems. This paper did not review studies on captive-born animals. Adjustment to captivity has been reported for some physiological systems in some species. However, for many species, permanent alterations to physiology may occur with captivity. For example, captive animals may have elevated GCs and/or reduced reproductive capacity compared to free-living animals even after months in captivity. Full adjustment to captivity may occur only in some species, and may be dependent on time of year or other variables. We discuss some of the methods that can be used to reduce chronic captivity stress.

## Teaser

Lay summary:

A review that compares changes in body mass, glucocorticoid and sympathetic responses, and reproductive and immune function, in wild animals recently introduced into captivity to their wild counterparts. Conclusion is that captivity can be a powerful chronic stressor that may be possible to mitigate, but the impact is highly species-specific.

## Introduction

The tens of thousands of vertebrate species on this planet are adapted to every condition from the Arctic to the tropics and from the mountain tops to the ocean depths. For all species, the environment contains both predictable changes (e.g. day–night transitions or seasonal variation) and unpredictable, uncontrollable threats to homeostasis and survival (Romero and Wingfield, 2016). Vertebrates have evolved a suite of defenses against the myriad unpredictable ‘shocks that flesh is heir to’ (Shakespeare, *Hamlet*, 3.1)—a set of conserved physiological responses known as the ‘stress response’. While the stress response can help an animal survive a threatening event, if noxious conditions are repeating or unrelenting two physiological changes take place. First, the reactive scope of the animal shrinks thereby decreasing the animal’s ability to cope (Romero *et al*., 2009). Second, the stress response itself can begin to cause physiological problems, a condition known as ‘chronic stress’. Even though there is no generally agreed upon definition of chronic stress or the time-frame of its onset, long-term stressor exposure or chronic stress, can lead to weight loss, immunosuppression, reproductive failure and psychological distress (Sapolsky *et al*., 2000). Because the stress response occurs when situations are perceived as threatening, regardless of whether the animal is experiencing physical damage, a drastic change of conditions can lead to symptoms of chronic stress even when the animal is unharmed. Consequently, when a wild animal is brought into captivity for the first time, symptoms of chronic stress can occur even though the physical needs of the animal are attended to.

In captivity, animals are provided with shelter and ample food. Nevertheless, captivity can often result in negative physiological outcomes, particularly for newly-captured animals. The conditions of captivity can be perceived as threatening, and if the perceived threat does not decrease, symptoms associated with chronic stress may result. The sources of stress in captivity are many, including cage restraint, human presence, an unfamiliar environment, and other, more subtle stressors, such as artificial light conditions (reviewed in Morgan and Tromborg, 2007). When wild animals are newly brought into captivity, it is frequently for research, conservation, agriculture (e.g. fisheries) or the exotic animal trade. To keep these animals healthy, symptoms of chronic stress should be minimized or eliminated. It is often assumed that with time, animals will adjust to captivity conditions and stress will disappear. Indeed, many animals seem to thrive in captivity. Unfortunately, many other species do not (Mason, 2010). In this review, we surveyed the literature to answer the following two questions: do wild animals eventually adjust to captivity conditions? And if so, how long does the period of adjustment typically take? This literature survey exclusively addressed wild animals introduced to captivity and not animals born in captivity.

We focused on several aspects of physiology that may be particularly affected by long-term stressor exposure. The acute stress response involves many behavioral and physiological changes, including activation of two hormonal pathways. The adrenomedullary response occurs within seconds of the onset of a stressor (Romero and Wingfield, 2016). The catecholamine hormones epinephrine and norepinephrine are rapidly released from the adrenal medulla. These cause an increase in heart rate, as well as an increase in muscle tone, an increase in blood pressure and other physiological and behavioral changes that enable an animal to survive a sudden stressor, such as a predator attack. The second hormonal response is initiated within minutes of the onset of a stressor, when a hormonal cascade triggers the synthesis and release of glucocorticoids (GCs)—steroid hormones that have wide-ranging effects on the body (Romero and Wingfield, 2016). While baseline levels of GCs help regulate metabolism, increased levels trigger an ‘emergency life history stage’, (Wingfield *et al.*, 1998), where resources and behaviors are directed towards survival of the crisis and away from long term investments. GCs have a strong impact on the immune and reproductive systems (Sapolsky *et al*., 2000). In this review, we focus on captivity’s effects on mass (one of the best-documented outcomes of chronic stress), GC concentrations and the immune, reproductive and adrenomedullary systems. We also document how the adjustment to captivity is impacted by time of year and how captivity effects persist after release. Finally, we discuss some of the ways that captivity stress may be mitigated.

## Methods

We surveyed the literature and gathered studies that compared wild-caught animals as they adjusted to captivity. We conducted a literature search through Web of Science using the search terms ‘captivity’ and ‘stress’ and ‘physiology’ or ‘endocrinology’ and related words. Because many papers reported on aspects of the stress response on animals that were in captivity but did not examine the effects of captivity itself, we were unable to devise search terms that included the studies we were interested in but excluded other research on stress in wild animals. We therefore devised the following criteria to determine whether papers should be included: (i) wild species were brought into captivity and physiological variables measured over the days to months of adjustment to captive conditions OR (ii) wild-caught captive animals were compared to free-living conspecifics AND (iii) the total captivity duration was at least 3 days (we did not include the many studies that measure only the acute stress effects of capture in the first 30 min to 48 hours). We further excluded two broad types of studies. One, we excluded studies where we could determine that all captive animals were captive-bred, as we were specifically interested in how well wild animals can adjust to captive conditions when taken from the wild (though we included some studies where the origin of captive animals was unclear). Second, we excluded studies of wild animals undergoing rehabilitation because it is not possible to distinguish between responses to captivity and responses to clinical interventions in animals that were injured or sick at capture. Once we had created a list of papers, we also checked the cited references of these studies for any important works our search terms missed.

There are many studies that focused on behavioral changes in captivity. However, the variables measured can be quite species-specific and difficult to interpret in a context of stress. Although we recognize the importance of behavior for the welfare of wild animals (reviewed in McPhee and Carlstead, 2010), we limited our focus to studies that included some physiological measurements (e.g. weight changes, hormone concentrations or immune measurements).

**Figure 1:**
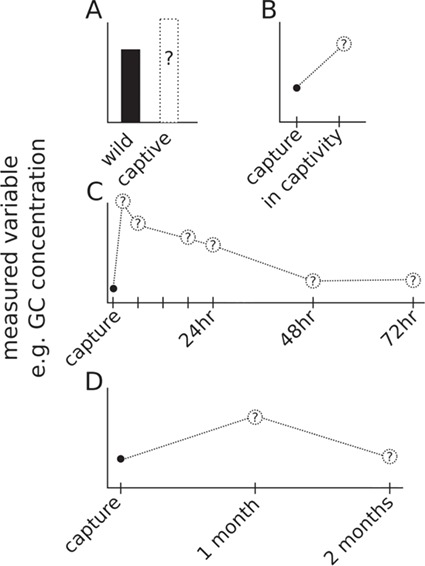
Examples of experimental designs to assess the effects of captivity on a physiological variable (e.g. GC concentration) (**A**) Comparison of captive individuals to free-living populations. In some cases, the free-living samples were acquired at the same time that the study population was brought into captivity. In other designs, the free-living samples were taken from entirely different populations than the origin of the captive animals (e.g. comparing zoo-housed animals to wild conspecifics). (**B**) Each individual measured immediately at capture and again after a period of captivity (days to months). (**C** and **D**) Each individual measured immediately at capture and resampled at multiple timepoints. Some studies focused on the first few days, with sampling points relatively close together (C). Other studies may not have taken another sample until several weeks after capture (D).

We found little standardization in experimental design in the papers examining the effect of captivity on physiology. We visually summarize the four most common experimental designs in Fig. 1. Many researchers compared animals that had been exposed to captivity (duration: 3 days to several years) to those that had not (Fig. 1A). In some cases, the free-living population was sampled when the captive population was initially captured. This was often the case in species where only a single blood sample could be drawn from an individual. In other studies, the free-living population was sampled entirely separately from the captive group. This was often the case for long-term captives, such as zoo-housed animals. Another common technique was to take a single pre-captivity sample and a single post-captivity sample on the same animal (duration of captivity 5 days to 3 months) (Fig. 1B). Other researchers used repeated sampling techniques—either sampling the same individual multiple times, or keeping different individuals in captivity for different durations before sampling. Some focused narrowly on the first few days of captivity (Fig. 1C), while others did not take a second sample until several weeks had passed (Fig. 1D). Furthermore, captive conditions varied between studies, with some studies bringing animals into closed indoor situations, whereas others placed captive animals into open outdoor pens. We considered each situation to represent captivity, but we were not able to contrast any differences in responses.

**Figure 2:**
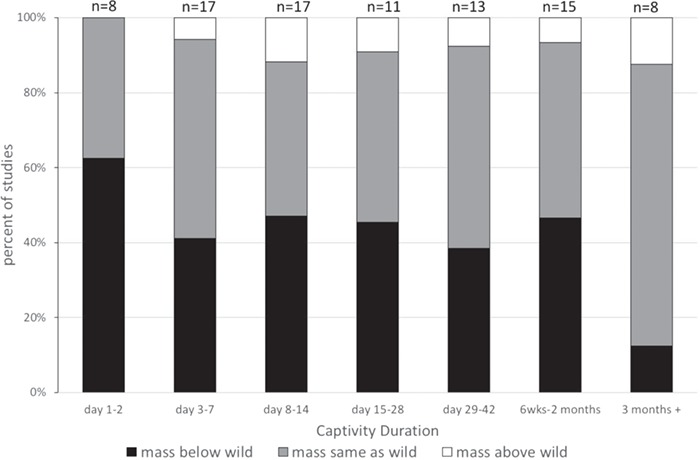
Weight change as a function of captivity duration. Data were collected from 35 studies listed in Table 1, with studies counted multiple times if they measured multiple time points after introduction to captivity. The number of species that lost weight in captivity (relative to wild, free-living animals) decreased with captivity duration.

**Figure 3:**
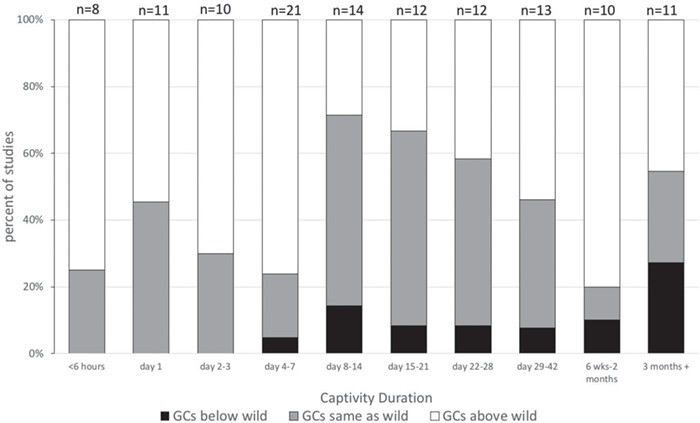
Change in baseline or integrated GCs as a function of captivity duration. Data were collected from the 47 studies listed in Table 3 that had a well-defined wild baseline value (i.e. plasma samples were collected within minutes of capture; fecal or urine samples were collected shortly after capture), with studies counted multiple times if they measured multiple time points after introduction to captivity. This figure does not include studies with seasonal effects on the GC response to capture.

**Figure 4:**
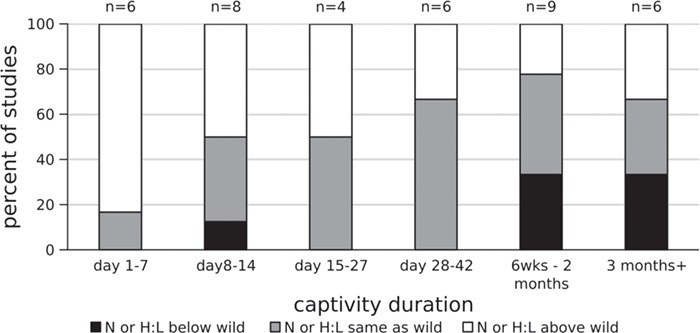
Changes in neutrophil or heterophil (N or H:L) to lymphocyte ratio in captivity as a function of time. Data were collected from 19 studies listed in Table 4, with studies counted multiple times if they measured multiple time points after introduction to captivity. The percent of studies that recorded elevated N or H:L ratio in captivity decreased with the amount of time spent in captivity.

We created summary figures for the trends we observed in weight, GC hormones and the immune system with respect to captivity duration (Figures 2–4). To construct these, we tallied the total number of studies that reported on the variable for a particular time window and determined whether the variable was above, below or equal to what it was in a free-living population. If a single report showed two different patterns (e.g. males and females had different patterns or two species were reported in the same paper), each pattern of was included separately. Therefore, one ‘study’ might be included multiple times in the figure. This also holds true for reporting patterns in the literature in the text and in the tables—if one paper reported multiple patterns in different groups of individuals, it was included more than once in calculating percentages of studies and was given more than one line on the tables. We did not include studies in the figures if there were marked seasonal differences in one species (see Section 9 for seasonal differences).

Because most of the papers we collected did not report effect sizes, a formal meta-analysis was not possible. Consequently, we focused on qualitative differences.

### Mass and body condition in captivity

After being brought into captivity from the wild, animals frequently experience a period of weight loss (Table 1). In 64% of studies (23 of 36), there was a documented decrease in mass associated with captivity during at least the initial capture period. Weight loss in captivity is likely to be attributable to chronic stress. Captive animals are not calorically restricted (as long as they choose to eat), which is not always the case in the wild, and they are not likely to use as many calories because cage restraint limits the amount of exercise that an animal can get in a day. Experimentally induced chronic stress has been demonstrated to lead to weight loss in mammals (e.g. Flugge, 1996), birds (e.g. Rich and Romero, 2005) and fish (e.g. Peters et al., 1980). In fact, weight loss is the most consistently seen effect of chronic stress (Dickens and Romero, 2013).

In 39% of studies where animals lost weight (9 of 23), the animals eventually regained the weight they had lost. In some cases, weight loss may be very transitory and last only a couple of days. For example, North Island saddlebacks (a bird native to New Zealand) lost weight on the first day of captivity, but by Day 3, they had not only regained weight, they were heavier than they were at capture (Adams *et al*., 2011). Transitory weight loss may be related to adjustment to the captive diet and not to major physiological problems. In other species, it may take weeks or months to regain the lost mass. House sparrows lose weight by Day 5–7 of captivity (Lattin *et al*., 2012; Fischer and Romero, 2016). In a long-term study of the species, they did not regain the weight they had lost for nearly 5 weeks (Fischer *et al*., 2018). Similarly, female possums lost weight for 5 weeks before beginning to gain again, and although they were kept for 20 weeks, they never fully recovered their lost weight (Baker *et al*., 1998). In 61% of studies (14 of 23), weight that was lost was never regained, though the studies may not have been long enough for weight to stabilize.

In some cases, weight loss depended on the characteristics of the animal at capture. For example, female possums lost weight over the first 5 weeks of captivity but some males gained weight during that period (Baker *et al*., 1998). When curve-billed thrashers were captured, birds from urban environments had higher body condition than desert birds, but after 80 days in captivity, their body conditions had converged to an intermediate value (Fokidis *et al*., 2011). Captivity may impact individuals differently depending on sex, population of origin or other individual characteristics, including transitory physiological states. (See  Section 9 for the effects of time of year on the ability to adjust to captivity.)

**Table 1:** Mass changes with captivity in wild animals

**Changes in mass during adjustment to captivity**	**Species**		**Study design**	**Timeframe**	**Citation**
Weight gain in captivity	Mammals	Steller sea lions (*Eumetopias jubatus*)	Repeated measures; pre- vs post-captivity	Average 2 months	(Mellish *et al*., 2006)^*^
		Richardson’s ground squirrel (*Urocitellus richardsonii*)	Repeated measures; multiple timepoints	14 days	(Hare *et al*., 2014)^*^
		Columbian ground squirrel (*Spermophilus columbianus*)	Repeated measures; multiple timepoints	13 days	(Bosson *et al*., 2009)^*^
		Brushtail possums (*Trichosurus volpecula*)	Repeated measures; multiple timepoints	37 days	(Day and O'Connor, 2000)^*^
	Birds	Ruff (*Philomachus pugnax*)	Repeated measures; multiple timepoints	Up to 1 year (mass increase in first few weeks, then seasonal fluctuations)	(Piersma *et al*., 2000)^*^
		North Island saddlebacks (*Philesturnus rufusater*)	Repeated measures; multiple timepoints	3 days	(Adams *et al*., 2010)^*^
No change in mass with captivity	Mammals	Vervet monkeys (*Chlorocebus aethiops*)^1^	Repeated measures; multiple timepoints	8 months	(Kagira *et al*., 2007)
		Brushtail possums (*Trichosurus volpecula*) (♂ only)	Repeated measures; multiple timepoints	20 weeks	(Baker *et al*., 1998)^*^
		Brushtail possums (*Trichosurus volpecula*) (♂)	Repeated measures; multiple timepoints	8 weeks	(Begg *et al*., 2004)^*^
	Birds	Curve-billed thrasher (*Toxostoma curverostre*)	Repeated measures;multiple timepoints	80 days	(Fokidis *et al*., 2011)^*^
	Reptiles	Duvaucel’s gecoks (*Hoplodactylus duvaucelli*)	Captive vs free-living population	>1 year	(Barry *et al*., 2010)^*^
	Amphibians	Cururu toad (*Rhinella icterica*)	Repeated measures; pre- vs post-captivity	13 days	(de Assis *et al*., 2015)^*^
		Toad (*Rhinella icterica*)	Captive vs free-living population, multiple timepoints^2^	up to 90 days	(Titon *et al*., 2018)^*^
Weight loss in captivity	Mammals	Beluga whale (*Delphinapterus leucas*)	Repeated measures; pre- vs post-captivity	10 weeks	(St Aubin and Geraci, 1988)^*^
		Harbor seal (*Phoca vitulina*) (juveniles)	Captive vs free-living populations^3^	>4 weeks	(Trumble *et al*., 2013)^*^
		African green monkey (*Cercopithecus aethiops*)	Multiple timepoints; different individuals	45 days	(Suleman *et al*., 2004)^*^
		Bighorn sheep (*Ovis canadensis canadensis*)	Repeated measures;multiple timepoints	14 days	(Franzman and Thorne. 1970)^*^
	Birds	Zebra finches (*Taeniopygia guttata*)	Captive vs free-living population	60 days^4^	(Ewenson *et al*., 2001)^*^
		Rufous-collared sparrows (*Zonotrichia capensis*)	Captive vs free-living population	2 weeks	(Ruiz *et al*., 2002)^*^
		Great tit (*Parus major*)	Repeated measures; pre- vs post-captivity	1 week	(Krams *et al*., 2013)^*^
		House sparrow (*Passer domesticus*)	Repeated measures; pre- vs post-captivity	1 week	(Fischer and Romero, 2016)^*^
		House sparrow (*Passer domesticus*)	Repeated measures; pre- vs post-captivity	1 week	(Fischer *et al*., 2018)^*^
		House sparrow (*Passer domesticus*)	Repeated measures; pre- vs post-captivity	5 days	(Lattin *et al*., 2012)^*^
		House sparrow (*Passer domesticus*)	Repeated measures; multiple timepoints	27 days	(Gormally *et al*., 2019)^*^
		House sparrow (*Passer domesticus*)	Repeated measures; multiple timepoints	66 days	(Love *et al*., 2017)^*^
	Amphibians	Toad (*Rhinella schneideri*)	Repeated measures; pre- vs post-captivity	60 days	(Titon *et al*., 2017)^*^
	Fish	Electric fish (*Gnathonemus petersii*)	Repeated measures; multiple timepoints	37 days	(Landsman, 1993)^*^
Weight lost then regained in captivity	Mammals	Rhesus macaques (*Macaca mulatta*)	Repeated measures; multiple timepoints	Weight decreased by Week 5, increased through 1 year	(Lilly *et al*., 1999)^*^
		European wild rabbits (*Oryctolagus cuniculus*)	Repeated measures; pre- vs post-captivity (different durations)	Weight decreased by Week 2, increased and stabilized by Week 4^5^	(Calvete *et al*., 2005)^*^
		Brushtail possums (*Trichosurus volpecula*) (♀ only)	Repeated measures; multiple timepoints	Weight decreased over 5 weeks, increased through Week 20	(Baker *et al*., 1998)^*^
		Tuco-tuco (*Ctenomys talarum*)	Repeated measures; multiple timepoints	Weight loss on Days 10 and 20, regained by Day 30	(Vera *et al*., 2011)^*^
	Birds	White-crowned sparrow (*Zonotrichia leucophrys*)	Repeated measures; multiple timepoints	Weight loss at Day 1, increased through day 14	(Wingfield *et al*., 1982)^*^
		Greenfinch (*Chloris chloris*)	Captive vs free-living population	Birds lighter at 1 month, heavier than wild at 2 months	(Sepp *et al*., 2010)^*^
		House sparrow (*Passer domesticus*)	Repeated measures; multiple timepoints	Weight loss on Days 11–25, regained by day 35	(Fischer *et al*., 2018)^*^
		Chukar partridge (*Alectoris chukar*)	Repeated measures; multiple timepoints	Weight loss at Day 1, partially regained at 5 and 9 days	(Dickens *et al*., 2009b)^*^
	Fish	Skipjack tuna (*Katsuwonus pelamus*)	Repeated measures; multiple timepoints	Weight loss at Day 2, regained at 20 days	(Bourke *et al*., 1987)
		Chukar partridge (*Alectoris chukar*)	Repeated measures; multiple timepoints	Weight loss at Day 1, partially regained at 5 and 9 days	(Dickens *et al*., 2009b)^*^
	Fish	Skipjack tuna (*Katsuwonus pelamus*)	Repeated measures; multiple timepoints	Weight loss at Day 2, regained at 20 days	(Bourke *et al*., 1987)

^1^No at-capture values—first measured at 2 months.

^2^Low sample sizes at each time point.

^3^Captive pups were rehabilitated after rescue.

^4^Slight weight loss from Day 10 to Day 60.

^5^Females did not reach at capture weight, but all spontaneously aborted or gave birth.

^*^Data from this paper were used to generate Fig. 2.

Weight loss was not the only pattern seen in captivity. In 17% of studies (6 of 36), animals gained mass above their starting condition. Some animals may benefit from the increased calories available in captivity and be able to maintain their weight. In other animals, however, *ad libitum* access to food and limits to exercise may cause them to become obese and face the myriad negative consequences of a high body mass or body fat content (West and York, 1998). In a study of domesticated budgerigars, birds were given *ad libitum* food and confined to cages that limited exercise. High body mass at the end of 28 days correlated with more DNA damage (Larcombe *et al*., 2015).

We visually summarized the patterns of weight changes in Fig. 2. We graphed the total percent of studies that showed weight gain, weight loss or no change in weight at different time points after introduction to captivity. There were no studies that recorded weight gain in the first day. Most weight gain seems to be reported at 15–28 days of captivity (38% of studies showed weight gain in that window). The percent of studies reporting weight loss decreased with increasing captivity duration, reflecting the fact that many studies show eventual regain of lost weight. This suggests that for many species where weight was lost, it would eventually be regained.

It is possible that seasonal fluctuations in weight may interfere with the assumptions that weight gain or loss is due to captivity. Captive ruffs and red knots have strong seasonal weight fluctuations in captivity associated with weight gain for migration and breeding (Piersma et al., 2000). If semi-naturalistic conditions are maintained in captivity (for example, if the animals are exposed to natural day length or are housed outdoors), then they may continue to experience seasonal weight changes that are not due to overfeeding or to long-term stressor exposure.

### Changes in GCs during the adjustment to captivity

One of the most common variables to measure when assessing the stress of captivity was GC concentrations. GC hormones (primarily cortisol in fish and most mammals; primarily corticosterone in reptiles, birds, amphibians, and rodents) are produced in the adrenal cortex, have multiple roles throughout the body, and can influence many other physiological systems. Acute stressors cause a transitory increase in GCs, which is eventually brought down by negative feedback. Long-term stressor exposure frequently results in changes in GC regulation, although the part of the GC response affected (baseline concentrations, stress-induced concentrations, or negative feedback) and the direction of the change are different in different species and circumstances (Dickens and Romero, 2013).

GCs can be assessed in several ways (Sheriff et al., 2011). The most common method is to measure circulating plasma GCs by taking a blood sample. The sampling procedure itself can cause an increase in GCs, so researchers usually try to acquire the first sample as quickly as possible—within 3 minutes of capture or disturbance is generally considered a good guideline (Romero and Reed, 2005). In many studies, it was not possible for the researchers to meet this standard because of the difficulty of capturing and bleeding the animals. In addition, some papers were written before the 3-minute standard had been established. It is also possible to assess GCs through other means. Fecal samples can be collected to measure metabolized GCs. Fecal samples provide an integrated profile of GC secretion over several hours to several days, depending upon the species, and reflect both baseline GCs and acute stress events (Wasser *et al*., 2000). Fecal sampling is convenient for many species when rapid capture and blood sampling is impractical. If the first fecal sample is collected soon after capture, it will not reflect the stress of captivity and may be considered a good free-living reference. Some researchers also used urinary GC metabolites, particularly in amphibian species, where animals could be left alone in a container of water from which excreted steroids were measured.

The initial capture and handling of wild animals is expected to cause an increase in circulating GC levels (an acute stress response). While some researchers investigated captivity-induced changes in the acute stress response itself (e.g. taking a plasma sample after a standardized 30-minute restraint stress at capture and again after a period in captivity), others incorporated the acute response to capture in the same analysis as longer-term captivity effects (e.g. taking a sample at 0, 2, 6, 18, 24, 48 and 72 hours post capture). Because of the variety of different measures used, we focused particularly on the captivity effects on baseline and integrated GCs (Table 2). However, we will also discuss the effects of captivity on the acute stress response and negative feedback of GC production (Table 3). Some researchers looked for the effects of captivity at different times of year—we do not include those studies in our calculations or in Tables 2 and 3 (see Section 9).

**Table 2:** Patterns of change in baseline and integrated GCs when wild animals are brought into captivity (this table does not include studies where the pattern was different in different seasons—those studies may be found in Table 6)

**GC Pattern during adjustment to captivity**	**Species**		**Study design**	**Timeframe**	**How were free-living GCs established?**	**Sample type**	**Citation**
No effect on GCs over captivity period	Mammals	Degu (*Octodon degus*)	Captive vs free-living populations	>1 year	Free-living population	Plasma (<2 min)	(Quispe *et al*., 2014)^*^
		Brushtail possums (*Trichosurus volpecula*) (♂ only)	Repeated measures; multiple timepoints	20 weeks	None—first sample at week 1 of captivity	Plasma (<5 min)	(Baker *et al*., 1998)
		Brushtail possums (*Trichosurus volpecula*) (♂)	Repeated measures; multiple timepoints	Up to 8 weeks	None—unclear when first sample was obtained	Plasma (time not given)	(Begg *et al*., 2004)
		Harbor seal (*Phoca vitulina*) (juvenile)	Captive vs free-living populations	>4 weeks	Free-living animals	Plasma (wild: 60 min captive: < 10 min)	(Trumble *et al*., 2013)^*^
		Tuco-tuco (*Ctenomys talarum*)^1^	Repeated measures; multiple timepoints	30 days	At-capture measure	Plasma (<3 min)	(Vera *et al*., 2011)^*^
	Birds	European starling (*Sturnus vulgaris*)	Repeated measures; pre- vs post- captivity	4 weeks +	Feather grown in the wild	Feathers	(Fischer *et al*., 2017)
		Western screech owl (*Otus kennicottii*)	Captive vs free-living populations	>1 month	Free-living animals	Plasma (<5 min)	(Dufty and Belthoff, 1997)^*^
		House sparrow (*Passer domesticus*)	Multiple timepoints; different individuals	Up to 4 weeks	Free-living animals	Plasma (<3 min)	(Martin *et al*., 2011)^*^
	Reptiles	Tuatara (*Sphenodon punctatus*) (♂ only)	Captive vs free-living populations	Unknown	Free-living population	Plasma (<20 min)	(Tyrrell and Cree, 1994)
		Kutum (*Rutilus frisii kutum*)	Captive vs free-living populations	3 days	Free-living population	Plasma (<3 min)	(Nikoo *et al*., 2010)^*^
GCs elevated in captivity	Mammals	Canada lynx (*Lynx canadensis*)^2^	Captive vs free-living populations	Long term (unknown)^3^	Free-living population	FGMs	(Fanson *et al*., 2012)^*^
		Spider monkey (*Ateles geoffroyi yucatanensis*)	Captive vs free-living populations	Long term (unknown)^2^	Free-living population	FGMs	(Rangel-Negrin *et al*., 2009)^*^
		African wild dog (*Lycaon pictus*)	Captive vs free-living populations	Long term (unknown)^2^	Free-living population	FGMs	(Van der Weyde *et al*., 2016)^*^
		Grevy’s zebra (*Equus grevyi*)	Repeated measures; multiple timepoints	6 weeks	At-capture sample; free-living population	FGMs	(Franceschini *et al*., 2008)^*^
		White rhinos (*Ceratotherium simum*)	Repeated measures; multiple timepoints	75 days	At-capture sample	FGMs	(Linklater *et al*., 2010)^*^
	Birds	Curve-billed thrasher (*Toxostoma curverostre*)	Repeated measures; multiple timepoints	80 days	At-capture samples	Plasma (<3 min)	(Fokidis *et al*., 2011)^*^
		White-crowned sparrow (*Zonotrichia leucophrys*)	Captive vs free-living populations	35 days	Free-living population	Plasma (<1 min)	(Marra *et al*., 1995)^*^
		White-throated sparrow (*Zonotrichia albicollis*)	Captive vs free-living populations	35 days	Free-living population	Plasma (<1 min)	(Marra *et al*., 1995)^*^
		Blackbirds (*Turdus merula*)	Repeated measures; pre- vs post-captivity	22 days	At-capture sample	Plasma (<3 min)	(Adams *et al*., 2011)^*^
		House sparrow (*Passer domesticus*)	Repeated measures; multiple timepoints	7 days	At-capture sample	Plasma (<3 min)	(Fischer and Romero, 2016)^*^
		House sparrow (*Passer domesticus*)	Repeated measures; multiple timepoints	7 days	At-capture sample	Plasma (<3 min)	(Fischer *et al*., 2018)^*^
		House sparrow (*Passer domesticus*)	Repeated measures; pre- vs post-captivity (multiple seasons)	5 days	At-capture sample	Plasma (<3 min)	(Lattin *et al*., 2012)^*^
		House sparrow (*Passer domesticus*)	Repeated measures; multiple timepoints	66 days	At-capture sample	Plasma (<3 min)	(Love *et al*., 2017)^*^
		House sparrow (*Passer domesticus*)	Repeated measures; multiple timepoints	24 days	At-capture sample	Plasma (<3 min)	(Gormally *et al*., 2019)^*^
		Southern pied babblers (*Turdoides bicolor*)	Captive vs free-living populations	5 days	Free-living population	FGMs	(Jepsen *et al*., 2019)^*^
	Reptiles	Tuatara (*Sphenodon punctatus*) (♀ only)	Captive vs free-living populations	Unknown	Free-living population	Plasma (<20 min)	(Tyrrell and Cree, 1994)^*^
		Garter snake (*Thamnophis elegans*)	Repeated measures; multiple timepoints	4 months	At-capture sample; free-living population	Plasma (<10 min)^4^	(Sparkman *et al*., 2014)^*^
		Tree lizard (*Urosaurus ornatus*)	Multiple timepoints; different individuals	Up to 3 weeks	At-capture samples	Plasma (<1 min)	(Moore *et al*., 1991)^*^
		Water snake (*Nerodia sipedon*)	Repeated measures; pre- vs post-captivity	5–8 days	At-capture samples	Plasma (<5 min)	(Sykes and Klukowski, 2009)^*^
		Brown treesnake (*Boiga irregularis*)	Multiple timepoints; different individuals	3 days	Free-living population	Plasma (<8 min)	(Mathies *et al*., 2001)^*^
	Amphibians	Cururu toad (*Rhinella icterica*)	Repeated measures; pre- vs post-captivity	3 months	At-capture sample	Plasma (<3 min)	(de Assis *et al*., 2015)^*^
		Toad (*Rhinella schneideri*)	Repeated measures; pre- vs post-captivity	60 days	At-capture sample	Plasma (<3 min)	(Titon *et al*., 2017)^*^
		Green frog (*Rana esculenta*)	Repeated measures; multiple timepoints (multiple seasons)	3 days	At-capture sample	Plasma (<5 min)	(Zerani *et al*., 1991)^*^
	Fish	Coral reef fish (*Hemigymnus melapterus*)	Captive vs free-living populations	2.5 months	Free-living population	Plasma (<6 min)	(Grutter and Pankhurst, 2000)^*^
		Wedge sole (*Dicologoglossa cuneate*) (juvenile)	Multiple timepoints; different individuals	45 days	At-hatching samples	Whole-body (time not given)	(Herrera *et al*., 2016)^*^
GCs increase at-capture, then decrease to approach wild baseline	Mammals	Beluga whale (*Delphinapterus leucas*)	Repeated measures; multiple timepoints	Peak:1 day approach free-living by 4 days	At-capture sample and free-living population	Plasma (time not given)	(St Aubin and Geraci, 1989)^*^
		Chacma baboon (*Papio ursinus*)	Repeated measures; multiple timepoints	Peak: 4 weeks approach long-term captives by 7 weeks	None—used long term captives as baseline.	Plasma (time not given)	(Steyn, 1975)
		African green monkey (*Cercopithecus aethiops*)	Multiple timepoints; different individuals	Peak: 1 day approach free-living by 2 days	Free-living population	Plasma (time not given)	(Suleman *et al*., 2004)^*^
		Mouse lemur (*Microcebus murinus*)	Repeated measures; multiple timepoints	Peak: 2 days approach free-living by 4 days	At-capture sample	FGMs	(Hamalainen *et al*., 2014)^*^
		Richardson’s ground squirrel (*Urocitellus richardsonii*)	Repeated measures; multiple timepoints	Peak: 3–5 days approach free-living by 6 days	At-capture sample	FGMs	(Hare *et al*., 2014)^*^
		Bottlenose dolphin (*Tursiops truncatus*)	Repeated measures; multiple timepoints	Peak: 1 day approach long-term captive by 2 weeks	Long-term captives	Plasma (time not given)	(Orlov *et al*., 1991)
	Birds	House sparrow (*Passer domesticus*)	Repeated measures; multiple timepoints	Peak: Day 7 approach free-living by Day 11	At-capture sample	Plasma (<3 min)	(Fischer *et al*., 2018)^*^
		House sparrow (*Passer domesticus*)	Repeated measures; multiple timepoints	Peak: Days 1–2 approach free-living by 1 month	At-capture sample	Plasma (<3 min)	(Kuhlman and Martin, 2010)^*^
		White-crowned sparrow (*Zonotrichia leucophrys*)	Repeated measures; multiple timepoints	Peak: Days 1–2 approach free-living by Day 14	At-capture sample	Plasma (time not given)	(Wingfield *et al*., 1982)^*^
	Reptiles	Skink (*Egernia whitii*)	Repeated measures; multiple timepoints	Peak: 1 day–1 week approach free-living by 4 weeks	At-capture sample	Plasma (<1 min)	(Jones and Bell, 2004)^*^
	Amphibians	Water frog (*Rana esculenta*)	Multiple timepoints; different individuals	Peak: Day 1 approach free-living by Day 7	Free-living populations	Plasma (<3 min)	(Gobbetti and Zerani, 1996)^*^
		Cane toad (*Rhinella marina*)	Repeated measures; multiple timepoints	Peak: Day 5 approach free-living by Day 12	At-capture sample	Urine	(Narayan *et al*., 2011)^*^
		Cane toad (*Rhinella marina*)	Repeated measures; multiple timepoints	Peak: Day 4 approach free-living by Day 14	At-capture sample	Urine	(Narayan *et al*., 2012)^*^
		Fijian ground frog (*Platymantis vitiana*)	Repeated measures; multiple timepoints	Peak: Day 5 approach free-living by Day 25	At-capture sample	Urine	(Narayan and Hero, 2011)^*^
	Fish	Flounder (*Paralichthys orbignyanus*)	Multiple timepoints; different individuals	Peak: 1 hour approach free-living by Day 1	Free-living animals	Plasma (<7 min)	(Bolasina, 2011)^*^
		Kahawai (*Arripis trutta*)	Multiple timepoints; different individuals	Peak: 2–3 hours approach free-living by Day 3	Free-living animals	Plasma (<4 min)	(Davidson *et al*., 1997)^*^
GCs lower in captivity	Mammals	Harbor porpoise (*Phocoena phocoena*)	Captive vs free-living populations	Long term (unknown)	Free-living population	Plasma (time not given)	(Siebert *et al*., 2011)^*^
		Gilbert’s potoroo (*Potorous gilbertii*)	Captive vs free-living populations	Long term (unknown)	Free-living population	FGMs	(Stead-Richardson *et al*., 2010)^*^
		Harbor seal (*Phoca vitulina*)	Repeated measures; multiple timepoints	Long term (unknown)	Free-living population	Plasma (time not given)	(Gardiner and Hall, 1997)^*^
		Black rhino (*Diceros bicornis*)^5^	Repeated measures; multiple timepoints	60 days	At-capture sample	FGMs	(Linklater *et al*., 2010)^*^
		White whale (*Delphinapterus leucas*)	Repeated measures; multiple timepoints	11 days	Long-term captives	Plasma (time not given)	(Orlov *et al*., 1991)
	Birds	European starling (*Sturnus vulgaris*)	Captive vs free-living population	Unknown	Free-living population	FGMs	(Cyr and Romero, 2008)
		Chukar partridge (*Alectoris chukar*)	Repeated measures; multiple timepoints	9 days	At-capture sample	Plasma (<3 min)	(Dickens *et al*., 2009a)^*^
	Amphibians	Toad (*Rhinella icterica*)	Captive vs free-living population, multiple timepoints^6^	Decreased from Days 30 to 60	Free-living population	Plasma (<3 min)	(Titon *et al*., 2018)^*^
High initial GCs decrease over capture period	Mammals	Rhesus macaques (Macaca mulatta)	Repeated measures; multiple timepoints	Decreased from Day 1 to 1 year	None—first sample after unknown time in trap.	Plasma (<50 min)	(Lilly *et al*., 1999)
		Brushtail possums (*Trichosurus volpecula*) (♀ only)	Repeated measures; multiple timepoints	Decreased from Week 1 to 20	None—first sample at Week 1 of captivity	Plasma (<5 min)	(Baker *et al*., 1998)
		Meadow vole (*Microtus pennsylvanicus*)	Multiple timepoints; different individuals	Decreased from Day 1 to Day 70	None—first sample at Day 1	Plasma (<1 min)	(Olsen and Seabloom, 1973)
		Vicuñas (*Vicugna vicugna*)	Repeated measures; multiple timepoints	Decreased from at-capture to Day 12	None—first sample after stressful capture (time not given)	Plasma (time not given)	(Bonacic and Macdonald, 2003)
		Eurasian otter (*Lutra lutra*)^7^	Repeated measures; multiple timepoints	Decreased from Days 2–5 to Days 5–10	None—first sample at Days 2–5	Plasma (time not given)	(Fernandez-Moran *et al*., 2004)
	Birds	Red knot (*Calidris canutus*)	Repeated measures; multiple timepoints	Decreased from first sample to 2 years	None—first sample at day 70	Plasma (3–38 min)	(Piersma and Ramenofsky, 1998)
	Fish	Red gurnard (*Chelidonichthys kumu*)	Repeated measures; pre- vs post-captivity (different durations)	Decreased from first sample to 1 day	None—first sample after long line capture	Plasma (<2 min)	(Clearwater, 1997)
		Snapper (*Pagrus auratus*)	Multiple timepoints; different individuals	Decreased form at-capture to Day 2	None—first sample after long line capture	Plasma (<10 min)	(Pankhurst and Sharples, 1992)
		Sardine (*Sardina pilchardus*)	Multiple timepoints; different individuals	Decreased from at-capture to Day 2	None—first sample after seine capture	Plasma (~3 min)	(Marcalo *et al*., 2008)

^1^Cortisol results only.

^2^No difference in GCs in females pre-breeding—GCs elevated in both sexes during breeding season.

^3^Captive population may include some captive-raised individuals.

^4^Blood sampling took longer in some samples.

^5^GC spike in many animals during first 2 weeks, but then drops well below at capture levels.

^6^GCs increased in non-calling toads, but sample sizes low.

^7^Some animals treated with long-acting neuroleptic, which had no effect on GC levels, so values were pooled.

^*^Data from this paper are incorporated into Fig. 3.

**Table 3:** Patterns of change in stress-induced GCs and negative feedback with captivity in wild animals

GC pattern during adjustment to captivity	Species		Study design	Timeframe	How was free-living GCs established?	Sample type	Citation
No change in acute stress-induced GCs over captivity period	Mammals	Tuco-tuco (*Ctenomys talarum*)	Captive vs free-living populations	20 days	Free-living population	Plasma (30 and 60 min)	(Vera *et al*., 2011)
	Birds	Curve-billed thrasher (*Toxostoma curverostre*)	Repeated measures; multiple timepoints	80 days	At-capture samples	Plasma (30 min)	(Fokidis *et al*., 2011)
		Blackbirds (*Turdus merula*)	Repeated measures; pre- vs post-captivity	22 days	At-capture sample	Plasma (30 and 60 min)	(Adams *et al*., 2011)
		Western screech owl (*Otus kennicottii*)	Captive vs free-living populations	>1 month	Free-living animals	Plasma (6–10 min)	(Dufty Jr and Belthoff, 1997)
		House sparrow (*Passer domesticus*)^1^	Repeated measures; pre- vs post-captivity	5 days	At-capture sample	Plasma (30 min)	(Lattin *et al*., 2012)
		House sparrow (*Passer domesticus*)	Multiple timepoints; different individuals	Up to 1 month	At-capture sample	Plasma (60 min)	(Kuhlman and Martin, 2010)
		House sparrow (*Passer domesticus*)	Repeated measures; multiple timepoints	7 days	At-capture sample	Plasma (30 min)	(Fischer and Romero, 2016)^*^
		House sparrow (*Passer domesticus*)	Repeated measures; multiple timepoints	7 days	At-capture sample	Plasma (30 min)	(Fischer *et al*., 2018)^*^
		House sparrow (*Passer domesticus*)	Repeated measures; multiple timepoints	66 days	At-capture sample	Plasma (30 min)	(Love *et al*., 2017)
		White-crowned sparrow (*Zonotrichia leucophrys*)^2^	Repeated measures; multiple timepoints	Up to 1 year	Free-living population	Plasma (<30 min)	(Romero and Wingfield, 1999)
	Fish	Winter flounder (*Pseudopleuronectes americanus*)	Repeated measures; multiple timepoints	Up to 1 year	Free-living population	Plasma (60 min)	(Plante *et al*., 2003)
Acute stress-induced GCs reduced in captivity	Birds	Chukar partridge (*Alectoris chukar*)	Repeated measures; multiple timepoints	9 days	At-capture sample	Plasma (30 min)	(Dickens *et al*., 2009a)
		White-crowned sparrow (*Zonotrichia leucophrys*)^3^	Repeated measures; multiple timepoints	Up to 1 year	Free-living population	Plasma (30 min)	(Romero and Wingfield, 1999)
Acute stress-induced GCs increased in captivity	Mammals	Degu (*Octodon degus*)	Captive vs free-living populations	>1 year	Free-living population	Plasma (30 and 60 min)	(Quispe *et al*., 2014)
	Birds	White-crowned sparrow (*Zonotrichia leucophrys*)^4^	Repeated measures; multiple timepoints	Up to 1 year	Free-living population	Plasma (30 min)	(Romero and Wingfield, 1999)
	Reptiles	Water snake (*Nerodia sipedon*)	Repeated measures; pre- vs post-captivity	5–8 days	At-capture sample	Plasma (60 min)	(Sykes and Klukowski, 2009)
	Amphibians	Eastern red-spotted newt (*Notophthalmus viridescens*)^5^	Repeated measures; multiple timepoints	>1 year	Free-living population	Plasma (30 min)	(Berner *et al*., 2013)
Negative feedback strength decreased with captivity, then increased	Birds	Chukar partridge (*Alectoris chukar*)	Repeated measures; multiple timepoints	Neg. feedback reduced at Day 5; recovered at Day 9	At-capture sample	Plasma (90 min after DEX)	(Dickens *et al*., 2009b)
Negative feedback strength increased with captivity	Birds	House sparrow (*Passer domesticus*)	Repeated measures; pre- vs post-captivity	5 days	At-capture sample	Plasma 90 min after DEX)	(Lattin *et al*., 2012)
Negative feedback strength did not change with captivity	Birds	House sparrow (*Passer domesticus*)	Repeated measures; multiple timepoints	66 days	At-capture sample	Plasma 90 min after DEX)	(Love *et al*., 2017)

^1^SI GCs lower post captivity in early winter, but no change during any other time of year.

^2^Outside of breeding season and molt.

^3^During the breeding season.

^4^During the post-breeding/molting season.

^5^SI GCs higher post captivity in pre-breeding and breeding season, not in winter.

Captivity does not influence GCs in all species. In 17% (10 of 59) studies, there was no recorded difference in GCs during or after the captivity period compared to free-living levels. In most studies, however, captivity caused a change in baseline or integrated GCs. In 42% of studies (25 of 59), wild animals had increased GCs at the end of the capture period compared to concentrations in free-living animals (periods of 3 days to several years). Elevated GCs are traditionally interpreted as an indication that animals are chronically stressed. Experimentally induced chronic stress can often lead to elevated baseline GCs, although this is by no means a universal response (Dickens and Romero, 2013). Adrenal hypertrophy may be an underlying mechanism explaining the long-term elevation of GCs. For example, long-term captivity led to increased adrenal mass in African green monkeys (Suleman et al., 2004) and mouse lemurs (Perret, 1982). In nine-banded armadillos, 6 months of captivity (but not 3 months) caused adrenal changes similar to those after a harsh winter (Rideout et al., 1985) and in herring gulls 28 days of captivity led to adrenal lesions (Hoffman and Leighton, 1985).

However, many studies that reported elevated GC concentrations at the end of the captivity period may eventually have shown decreased GCs had the study been carried out for longer. For example, house sparrows had elevated baseline GCs after 1–7 days in captivity (Kuhlman and Martin, 2010; Lattin et al., 2012; Fischer and Romero, 2016). But when captive house sparrows were sampled repeatedly over 6 weeks of captivity, the high baseline GCs seen at Day 7 were dramatically reduced over Days 11–42 and approached at-capture concentrations in one study (Fischer *et al*., 2018), but did not decrease in another study (Love *et al*., 2017).

The duration of captivity in the studies we collected was quite variable, ranging from 3 days to several years. To consolidate the patterns from multiple studies with different sampling times, we graphed the percent of studies with elevated GCs (relative to free-living levels) against captivity duration (Fig. 3). We expected the percent of studies with elevated GCs to decrease as captivity duration increased (as shown in Fig. 1C and D). This pattern would indicate an adjustment to captivity conditions and is a typical *a priori* prediction in the literature. However, we found that 45% (5 of 11) of species continued to have elevated GCs after 3 months or more of captivity. This suggests that for many species, there is never a complete adjustment to captivity. It is also possible that a publication bias exists in the papers we collected. When researchers did not see a difference between long-term captives and free-living animals, they may have been less likely to publish, or perhaps included those results in other studies that did not appear in our literature searches. It is interesting to note that the fewest studies reported elevated GCs at around two weeks post captivity, the amount of time that many researchers allow for their study species to become acclimated to laboratory conditions (e.g. Davies et al., 2013; Lattin and Romero, 2014; McCormick et al., 2015).

The analysis in Fig. 3 contains data collected from many different taxa, study designs, etc. A more informative methodology to investigate how GCs change over time in captivity is to compare multiple timepoints within the same experiment. We found 38 studies that used repeated sampling. Researchers either repeatedly sampled individuals or captured many subjects at once and sampled them after different captivity durations. In study designs with repeated sampling, 42% of studies (16 of 38) showed an early increase in GCs followed by a decrease back to free-living levels (e.g. Fig. 1C and D, the *a priori* prediction for GC adjustment to captivity). Of the remaining studies, 32% (12 of 38) matched the pattern in Fig. 3 with no decrease in GC concentrations over time, 13% (5 of 38) showed decreased GC concentrations in captivity and 11% (4 of 38) reported no change in GCs whatsoever. When the expected peak and fall of GCs was observed, the timescale of adjustment to captivity varied. Baseline GCs in mouse lemurs returned to at-capture levels by Day 5 (Hamalainen *et al*., 2014) while the Fijian ground frog had elevated urinary GCs until Day 25 post capture (Narayan and Hero, 2011).

In some studies with repeated measures designs, the researchers did not or could not obtain a sample that represented free-living animals. In these cases, the first sample could not be acquired until minutes, hours or even days after capture. In all nine studies where this was the case (see Table 2), initially high concentrations of GCs decreased over the study period in at least some animals. This is consistent with the pattern we expect for animals successfully adjusting to captivity: capture, handling and the initial transfer to captivity result in high GCs that decrease as the animal adjusts. For example, female brushtail possums were not sampled until days after their capture and transfer to captivity, but showed decreasing plasma GCs from week 1 to week 20 of captivity (Baker *et al*., 1998).

These studies on baseline GCs together demonstrate a pattern wherein approximately half of species appear to adjust to captivity. Although some species seem to take longer to acclimate to captive conditions than others, it appears that many species will eventually show a reduction in GCs after an initial peak. We see this pattern across taxonomic groups, in birds, fish, reptiles, amphibians and mammals. However, we should be careful to not interpret a reduction in circulating baseline GCs, fecal GC metabolites or urinary GCs as a complete adjustment to captivity or an elimination of chronic stress. Even when baseline GCs have returned to free-living levels, other aspects of the animals’ physiologies may be negatively impacted. For example, even though circulating GCs were only elevated for 1 day in African green monkeys, adrenal mass was almost doubled after 45 days in captivity (Suleman *et al*., 2004). Similarly, while it is tempting to conclude that elevated GCs are diagnostic of chronic stress, it should be kept in mind that baseline GCs have many functions in metabolism and energy use. A change of baseline GCs in captivity could merely reflect a change in energy requirements and not the physiological damage we associate with chronic stress. Furthermore, a reduction in GCs in captivity, as seen in 14% of studies (8 of 59), could be interpreted as a reduction in allostatic load or as the exhaustion of adrenal capacity.

#### Impact of captivity on acute stress response and negative feedback of GC production

Relatively few researchers have explicitly investigated the effects of captivity on the acute GC stress response (see Table 3). Of those that have, 65% (11 of 17) found no effect of captivity (captivity duration 5–80 days). The six studies that reported changes in stress-induced GCs showed changes in opposite directions. In two studies, stress-induced GCs were decreased in captivity, even though the captive periods of 9 days (Dickens et al., 2009a) and 1 year (Romero and Wingfield, 1999) were quite different. In contrast, stress-induced GCs were increased in captivity in four studies over similar time frames. Three studies had animals in captivity for about a year (Romero and Wingfield, 1999; Berner *et al*., 2013; Quispe *et al*., 2014), with 5–8 days in the fourth study (Sykes and Klukowski, 2009).

The negative feedback of the GC response to stress, where high GC levels lead to the inhibition of GC production, is very important for the control of physiological stress (Vitousek *et al*., 2019). Although chronic stress has frequently been found to affect the negative feedback of GC production (Dickens and Romero, 2013), we found only three studies that explicitly measured negative feedback strength in animals immediately at capture and after a period of captivity. In each case, animals were injected with a synthetic GC (dexamethasone) after mounting a stress response to stimulate maximum negative feedback. The strength of negative feedback increased slightly in house sparrows after 5 days of captivity (Lattin *et al*., 2012), but in the same species showed no change after 21, 42 or 66 days (Love *et al*., 2017). In contrast, negative feedback strength decreased after 5 days of captivity in chukar partridges but returned to its at-capture strength by 9 days (Dickens *et al*., 2009b). This is an important aspect of stress physiology, one that is critical for the total amount of GC exposure, and warrants further study to determine whether it is impacted by the stress of captivity in many species.

### Immune consequences of captivity

Stress has well-documented, but sometimes complex, effects on the immune system. In large part, these changes are due to the acute or long-term effects of elevated GCs on leukocyte populations. GCs can cause immune redistribution, moving lymphocytes out of the bloodstream and into the skin, spleen and lymph nodes, where they will be available in case of a wound (Dhabhar and McEwen, 1997; Johnstone *et al*., 2012). GCs can also cause proliferation or mobilization of neutrophils (most vertebrates) or heterophils (birds and some reptiles) (Dale *et al*., 1975; Gross and Siegel, 1983; Johnstone *et al*., 2012). Together, these effects on leukocyte populations result in a change in the neutrophil or heterophil to lymphocyte ratio (N or H:L ratio) (Dhabhar and McEwen, 1997; Johnstone *et al*., 2012). A change in the N or H:L ratio does not necessarily mean that an animal’s immune system is hypo- or hyperactive. Instead, this acts as another metric similar to GC secretion. A long-term increase in N or H:L ratio, like a long-term increase in circulating GCs, can be an indication that an animal is suffering from chronic stress (Davis *et al*., 2008).

We summarized the 23 studies that reported leukocyte counts in Table 4. Although the N or H:L is a useful metric, in some studies the researchers chose to report total number or percent of different leukocyte types without calculating or performing statistics on the relative abundances of neutrophils/heterophils and lymphocytes. In these cases, we inferred the direction (or presence) of change after captivity of the N or H:L ratio based on the changes in leukocyte counts or percentages that were reported. In two studies, only the total number of leukocytes was reported without further subdivision of leukocyte types. In 48% of studies (10 of 21), N or H:L ratio was elevated at the end of the measured captivity duration relative to its free-living value. 29% of studies (6 of 21) documented no change in N or H:L ratio over the study period. N or H:L ratio was decreased in 24% of studies (5 of 21). In one study (in the Fijian ground frog), the N:L ratio was elevated for 15 days in captivity, but then returned to wild levels by Day 25, resulting in no overall change (Narayan and Hero, 2011). Kuhlman and Martin (2010) further investigated leukocyte redistribution to the skin in house sparrows, comparing Day 1of captivity to Day 30. They concluded that the changes in H:L ratio were not due to redistribution of leukocytes, at least in this instance. We summarized the overall patterns of N or H:L ratio compared to captivity duration in Fig. 4. The number of studies reporting an increase in N or H:L ratio decreases with captivity duration. This suggests that many or most species do adjust to captivity, and an initially high N or H:L ratio may decrease given sufficient time.

**Table 4:** Changes in leukocytes during captivity

**Species**		**Study design**	**How was free-living value established?**	**Timeframe**	**WBCs**	**H or N**	**L**	**H or N:L ratio**	**Citation**
Mammals	Spanish ibex (*Capra pyrenaica hispanica*)	Repeated measures; multiple timepoints	At-capture sample	14 months	↓	–	↓	↑ (n.c.)	(Peinado *et al*., 1995)
	Rhesus macaques (Macaca mulatta)	Repeated measures; multiple timepoints	At-capture sample	1 year	↓	↓	↑	↓ (n.c.)	(Lilly *et al*., 1999)
	Brushtailed possums (*Trichosurus vulpecula*)	Repeated measures; multiple timepoints	None—first sample at Week 1 of captivity	20 weeks	–			–	(Baker *et al*., 1998)
	Beluga whale (*Delphinapterus leucas*)	Repeated measures; multiple timepoints	At capture sample and free-living population	2.5 months	↑	↑	↓	↑ (n.c.)	(St Aubin and Geraci, 1989)
	brushtailed possums (*Trichosurus vulpecula*)	Repeated measures; multiple timepoints (different housing conditions)	None—unclear when first sample was obtained	2 months	–	–	–	– (n.c.)	(Begg *et al*., 2004)
	Howler monkey (*Alouatta caraya*)	Repeated measures; pre- vs post-captivity	At-capture sample	2 months	–	–	–	– (n.c.)	(Sanchez-Sarmiento *et al*., 2015)
	Steller sea lions (*Eumetiopias jubatus*)	Repeated measures; pre- vs post-captivity	At-capture sample and free-living population	2 months	↓				(Mellish *et al*., 2006)
	Black rhinoceros (*Diceros bicornis michaeli*)^1^	Repeated measures; pre- vs post-captivity	None – first sample after stressful capture (up to 1 hour)	3–4 weeks		↑	↓	↑ (n.c.)	(Kock *et al*., 1999)
	Vicuñas (*Vicugna vicugna*)^2^	Repeated measures; multiple timepoints	At-capture sample^3^	12 days	–	–	–	–	(Bonacic and Macdonald, 2003)
Birds	Red knots (*Calidris canutus*)	Captive vs free-living population	Free-living population	~1 year	–	↓	–	↓ (n.c.)	(Buehler *et al*., 2008)
	Ruff (*Philomachus pugnax*)	Repeated measures; multiple timepoints	None—does not say when first sample taken relative to capture	1 year	–				(Piersma *et al*., 2000)
	Greenfinches (*Chloris chloris*)	Captive vs free-living population	Free-living population	2 months	–	–	↑	↓	(Sepp *et al*., 2010)
	Zebra finches (*Taeniopygia guttata*)	Captive vs free-living population	Free-living population	10 days	↓			↓	(Ewenson *et al*., 2001)
				2 months	–			↓	
	House sparrow (*Passer domesticus*)^4^	Repeated measures; early- vs late-captivity	None—first sample 1–2 days in captivity	1 month	–	–	↓	↑ (n.c.)	(Kuhlman and Martin, 2010)
	Herring gull (*Larus argentatus*)	Repeated measures; multiple timepoints	At-capture sample	4 weeks	↑	↑	–	↑ (n.c.)	(Hoffman and Leighton, 1985)
	Rufous-collared sparrows (*Zonotrichia capensis*)	Captive vs free-living population	Free-living population	2 weeks		↑	↓	↑	(Ruiz *et al*., 2002)
Reptiles	Garter snakes (*Thamnophis elegans*)	Repeated measures; pre- vs post-captivity	At-capture sample and free-living population	4 months				↑	(Sparkman *et al*., 2014)
Amphibians	Cururu toad (*Rhinella icterica*)	Repeated measures; pre- vs post-captivity	At-capture sample	3 months	↑			–	(de Assis *et al*., 2015)
	Fijian ground frog (*Platymantis vitiana*)	Repeated measures; multiple timepoints	At-capture sample	15 days		↑	↓	↑	(Narayan and Hero, 2011)
				25 days		–	–	–	
	Mole salamanders (*Ambystoma talpoideum*)	Repeated measures; pre- vs post-captivity	At-capture sample	10 days		–	↓	↑	(Davis and Maerz, 2008)
Fish	Kutum (*Rutilus frisii kutum*)	Captive vs free-living population	Free-living population	3 days	↑	↑	↓	↑ (n.c.)	(Nikoo *et al*., 2010)

Timeframe refers to the longest duration of captivity measured. WBC = total white blood cells; H = heterophils; N = neutrophils; L = lymphocytes; n.c. = not calculated (in this case, a count or percentage of heterophils or neutrophils and lymphocytes was measured in the paper, but H or N:L ratio was not directly compared. Presence/direction of change in the rctypes); ↑ or ↓ = higher or lower than free-living; – = no change from free-living.

^1^Pattern only seen in rhinos translocated from high to low (not high to high) elevation.

^2^Total WBCs and N:L ratio also compared to free-living wild populations of a similar species—there was no difference.

^3^Comparison to values collected in another study and species (llamas and alpacas).

^4^Circulating leukocytes and skin-infiltrating leukocytes were measured. See text for skin leukocyte patterns.

Some studies also reported the total leukocyte counts, sometimes without further subdividing them into classes. While decreased circulating leukocytes has been associated with stress (generally because of redistribution rather than destruction of cells) (Dhabhar, 2002), there was no clear pattern with the number of leukocytes in captivity. 53% of studies (9 of 17) showed no change in total white blood cells compared to free-living animals by the end of the captivity period; 23.5% (4 of 17) showed a decrease in circulating leukocytes; and 23.5% (4 of 17) showed an increase (captivity duration 3 days to 1 year, see Table 4).

Importantly, neither total leukocyte numbers nor the N or H:L ratio provide a very strong indication of immune capacity. Some researchers have used more direct measurements of immune functionality. The bacterial killing assay is a way to determine how effectively fresh whole blood can eliminate bacteria. This assay has the advantage of determining the real effectiveness of the immune system against pathogens (Millet *et al*., 2007). In the cururu toad, whole blood was less effective at killing bacteria after 13 days of captivity (de Assis *et al*., 2015) and in two other toad species, killing capacity decreased by 60 but not 30 days (Titon *et al*., 2017, 2018). Similarly, in red knots held in captivity for 1 year, whole blood was less effective at eliminating two *Staphylococcus* species than in wild living birds (though there was no difference in *Escherichia coli* elimination) (Buehler *et al*., 2008). In contrast, there was an increased proportion of *E. coli* killed after 3 weeks of captivity in house sparrows (Love *et al*., 2017).

Another way to measure immune responsiveness is by measuring a proliferative response against non-specific antigens. In some studies, this is done by culturing a sample of blood along with an antigen and quantifying cell division. In male brushtail possums, the proliferative response to the plant toxin phytohemagglutinin decreased over 20 weeks but increased by 1 year (Baker *et al*., 1998). In female possums, the proliferative response increased from 11 to 15 weeks in captivity, and then remained at that high level for at least a year (Baker *et al*., 1998). In another study in male brushtail possums, leukocyte proliferation to a *Mycobacterium* protein derivative increased after 4 and 6 weeks of captivity, but only when the animals were housed in high-density pens to create crowding (Begg *et al*., 2004). The proliferative response to phytohemagglutinin can also be measured in-vivo if PHA is injected into the skin and the degree of swelling is quantified. In zebra finches, there was no difference in the *in vivo* PHA response between newly captured birds and those held for 10 or 16 days (Ewenson *et al*., 2001).

Two studies have attempted to quantify the strength of the adaptive immune system in captivity. In red knots, plasma was plated with rabbit red blood cells. The degree of hemolysis and hemagglutination provided a measure of complement and natural antibody action. Hemolysis and hemagglutination were similar in wild and captive birds when they were measured at the same time of year, which suggests that the strength of the adaptive immune response is unaffected by captivity (Buehler *et al*., 2008). Conversely, newly captured killifish had a stronger response to antigen after immunization than 4–6-week captives, suggesting that the adaptive immune system was less effective after captivity (Miller and Tripp, 1982).

Overall, there does not seem to be a single pattern for immune regulation with captivity. While captivity has been shown to repress immune function in some species (e.g. reduced bacterial killing in red knots and toads), in other species, the immune system may be hyperactivated. For example, in house sparrows, gene expression for pro-inflammatory cytokines was elevated in captive birds (2- and 4-week captives) compared to newly caught animals, which was interpreted as hyperinflammation in captive birds (Martin *et al*., 2011). Changes in the immune response with chronic stress are thought to be most strongly tied to GC release. However, the impacts of GCs on the immune system can be complex. In the short term, GCs typically induce an immune response, while they can be immunosuppressive over the long term, although these interactions tend to be context-dependent (Dhabhar and McEwen, 1997; Martin, 2009). As the interaction between GCs and immunity is complex and context specific, and as the interaction of GCs to captivity can be complex as well (see Changes in GCs during the adjustment to captivity), it is not currently possible to predict whether captivity conditions will result in appropriate or inappropriate immune activity. However, there has been limited work in this area.

### Effects of captivity on the reproductive system

Captivity has well-documented negative impacts on reproductive biology. In many species, captive breeding for research or conservation purposes can be a challenge. Even the house sparrow, so commonly used as a model species, does not readily breed in captivity (Lombardo and Thorpe, 2009). In 74% of studies (17 of 23), the transition to captivity resulted in reduced reproductive capacity in wild species (Table 5). Note, however, that these papers do not cover an extensive literature on captive breeding, including in individuals who have spent decades in captivity or were born in captivity, which is beyond the scope of this review. Here, we focus only on those papers that studied reproductive capacity of recent captives (only within the first year) and that examined a mechanism for reduced reproduction. There was no obvious taxonomic pattern for species that had reduced reproductive ability in captivity compared to those that had no documented reproductive problems. Duration of captivity did not appear to be a factor either. In one study of water frogs, reproduction in both males and females were negatively impacted by only 3 days of captivity (Zerani *et al*., 1991), while in jack mackerel, reproduction was inhibited after a full year of adjusting to captivity (Imanaga *et al*., 2014).

**Table 5:** Reproductive effects of captivity in wild animals (if multiple times of year were examined, only breeding season is included in this table)

**Hormonal changes during adjustment to captivity**	**Species**		**Study design**	**Timeframe**	**Variable measured**	**How were free-living state established?**	**Citation**
Reproductive capacity decreased in captivity	Mammals	White rhino (*Ceratotherium simum*)	Repeated measures; multiple timepoints	75 days	Fecal T (males) and Progestin (females)	At-capture samples	(Linklater *et al*., 2010)
		Black rhino (*Diceros bicornis*)	Repeated measures; multiple timepoints	60 days	Fecal T (males) and progestin (females)	At-capture samples	(Linklater *et al*., 2010)
		Mouse lemur (*Microcebus murinus*) (♀ only)	Pathology of dead captive animals	Variable—usually years in captivity	Histological examination of reproductive organs (follicular growth)	Reproductive pathology increased with captivity length	(Perret, 1982)
	Birds	Brown-headed cowbird (*Molothrus ater*) (♂ only)^1^	Captive vs free-living population	6 months +3 months photostimulation	Gonad size and plasma T	Free-living population	(Dufty Jr and Wingfield, 1986)
		House sparrow (*Passer domesticus*) (♂)	Repeated measures; multiple timepoints	3 months	Sperm production, beak color, testes size	At-capture samples and free-living population	(Lombardo and Thorpe, 2009)
	Reptiles	Brown treesnakes (*Boiga irregularis*) (♂)	Captive vs free-living population	4–8 weeks	Sexual maturity (testes development)	Free-living population	(Aldridge and Arackal, 2005)
		Anole lizard (*Anolis pulchellus*) (♀)	Multiple timepoints; different individuals	4 weeks	Plasma vitellogenin; ovary state^2^	Free-living population	(Morales and Sanchez, 1996)
		Tree lizard (*Urosaurus ornatus*) (♂)	Repeated measures; multiple timepoints	3 weeks	Plasma T	At-capture samples	(Moore *et al*., 1991)
		Snapping turtle (*Chelydra serpentina*)	Repeated measures; multiple timepoints	1 week	Plasma T^3^	At-capture sample	(Mahmoud *et al*., 1989)
	Amphibians	Water frog (*Rana esculenta*) (♂)	Repeated measures; multiple timepoints	2 weeks	Plasma T and E2	At capture samples	(Gobbetti and Zerani, 1996)
		Water frog (*Rana esculenta*)	Repeated measures; multiple timepoints	3 days	Plasma T and E2^4^	At capture sample	(Zerani *et al*., 1991)
		Toad (*Rhinella icterica*)	Captive vs free-living population, multiple timepoints	1 week	Plasma T	Free-living population	(Titon *et al*., 2018)
		Toad (*Rhinella schneideri*)	Repeated measures; multiple timepoints	60 days	Plasma T	At-capture sample	(Titon *et al*., 2017)
	Fish	Jack mackerel (*Trachurnus jabonicus*) (♀)	Captive vs free-living population	1 year	Egg maturity, reproductive stage, gnrh gene expression^5^	Free-living population	(Imanaga *et al*., 2014)
		Electric fish (*Gnathonemus petersii*)	Repeated measures; multiple timepoints	37 days	Sex-specific behaviors, plasma T and 11KT (males)	At capture samples	(Landsman, 1993)
		Sardine (*Sardina pilcardus*)	Captive vs free-living population	4 weeks	Gonadosomatic index	Free-living population	(Marcalo *et al*., 2008)
		Red gurnard (Chelidonichthys kumu) (♀)	Multiple timepoints; different individuals AND repeated measures; multiple timepoints	4 days	Plasma T, E2, egg development	Free-living population	(Clearwater, 1997)
No difference in reproductive capacity in captivity	Mammals	Armadillos (*Dasypus novemcinctus*) (♂)	Repeated measures; multiple timepoints	Up to 3 years	Plasma T	Free-living population	(Czekala *et al*., 1980)
	Birds	White-crowned sparrows (*Zonotrichia leucophrys*)^6^	Repeated measures; multiple timepoints	Up to day 20 or 33	Plasma LH, plasma T (males only)	At-capture sample	(Wingfield *et al*., 1982)
	Reptiles	Striped plateau lizard (*Sceloporus virgaltus*) (♀)	Repeated measures; multiple timepoints	Up to 3 months	Plasma P, T and E2^7^	Free living population	(Weiss *et al*., 2002)
		Skink (*Egernia whitii*) (♂)	Repeated measures; multiple timepoints	4 weeks	Plasma T	At-capture samples	(Jones and Bell, 2004)
		Brown treesnake (*Boiga irregularis*)	Multiple timepoints; different individuals	3 days	Plasma T and ovarian follicle development	Free-living population	(Mathies *et al*., 2001)
	Amphibians	Water frog (*Rana esculenta*) (♀)	Repeated measures; multiple timepoints	2 weeks	Plasma T and E2^8^	At capture samples	(Gobbetti and Zerani, 1996)

^1^Different photosimulation and social stimulation tested—maximal testicular regrowth (long days + females) still below wild, though in that group, T was the same as wild.

^2^Vitellogenin levels recovered by E2 use.

^3^T spikes during the first 24–48 hours of captivity, but decreases below at-capture levels.

^4^E2 spikes during first hours of captivity, but quickly decreases below at-capture levels.

^5^E2 higher in captive than wild.

^6^There was a transitory increase in LH at around Weeks 1–3 that came back to at-capture levels in multiple experiments.

^7^T lower in captivity, but only after egg-laying.

^8^E2 spike in first 6 hours of captivity but then returns to at-capture levels.

Different researchers measured different variables for reproductive capacity. Many studies analyzed reproductive steroid hormones (primarily testosterone in males and estrogen and/or progesterone in females). However, other variables were also measured, including gonad size and development, behavior and gamete development. In house sparrows, Lombardo and Thorpe (2009) found decreased sperm production, reduced testes size and a change in beak color from breeding-season black to wintering brown after 3 months of captivity. Female anole lizards experienced a rapid decrease in plasma vitellogenin (a protein necessary for yolk production) followed by regression of developing follicles (Morales and Sanchez, 1996). In electric fish, behavioral differences between males and females were reduced in captivity until they disappeared or even reversed. This occurred concurrently with decreases in testosterone and 11-ketotestosterone (a potent fish androgen) in males (Landsman, 1993).

The reduction of reproductive capacity might be tied to GC levels. GCs can be powerful suppressors of reproductive steroids (Sapolsky *et al*., 2000). Prolonged GC exposure can lead to decreased production of testosterone or estradiol, which can then have downstream effects on gonad development, egg maturation, sperm production and behavior. In green treefrogs, a decrease in sex steroids was concurrent with an increase in GCs (Zerani *et al*., 1991). However, in black rhinos, males had suppressed fecal testosterone and females had suppressed fecal progestins even though GC levels were below free-living levels for most of the captivity period (Linklater *et al*., 2010).

Captivity did not always result in suppression of reproduction but in most studies that did not show an effect of captivity, reproductive hormones were the only variables measured. The only exception was in the brown treesnake, where 3 days of captivity did not affect either testosterone or ovarian development (both were very low in free-living and captive animals) (Mathies *et al*., 2001). However, another study in brown treesnakes found underdeveloped testes in males after 4–8 weeks of captivity (Aldridge and Arackal, 2005). Captivity may affect sexual variables differently in males and females. For example, in water frogs held in captivity for 2 weeks, only males appeared to be negatively affected by captivity (Gobbetti and Zerani, 1996), which is opposite what is typically expected.

Overall, it appears that captivity tends to have a negative impact on reproduction in most species. However, there are relatively few studies that specifically examine the reproductive physiology of newly-captured animals. Furthermore, given that many animals eventually do breed in captivity while others do not, it is not clear how long-lasting these impacts may be or why they impact some species more than others.

### Adrenomedullary effects of captivity

The adrenomedullary arm of the stress response can be difficult to measure. Measuring epinephrine or norepinephrine in the blood is relatively straightforward, but these hormones increase within seconds of disturbance, meaning that acquiring a free-living baseline in a wild animal is difficult without substantial acclimation to human presence. We excluded most studies that measured epinephrine or norepinephrine, as sampling techniques between wild and captive animals differed in ways that would obscure the meaning of their results. For example, plasma norepinephrine under anesthesia (collected within 50 minutes) decreased over 19 months of captivity in rhesus macaques, though a free-living sample could not be obtained under the same conditions (Lilly *et a*l., 1999), and captive-raised bighorn sheep had a higher epinephrine response to a drop-net capture technique than did free-living sheep, though they had similar norepinephrine responses (Coburn *et al*., 2010).

Recording heart rate is another way to infer activity of the adrenomedullary system (Romero and Wingfield, 2016). Heart rate recordings typically involve the use of specialized and expensive equipment, but can give instantaneous updates on heart rate. In addition, scientists can measure heart rate variability, which gives a metric of how much relative control the sympathetic and parasympathetic nervous systems have over heart rate (Romero and Wingfield, 2016). However, depending on the type of heart rate recording device, it may be impossible to obtain baseline free-living heart rates. Although several researchers have had success measuring heart rate in free-living animals (e.g. white-eyed vireos; Bisson *et al*., 2009), to our knowledge, there has not yet been a study that directly compares heart rate in free-living and captive animals of the same species.

Heart rate has been measured in only a few species during the transition to captivity. In newly-captured bighorn sheep, heart rate during restraint and blood sampling decreased from Days 1–2 (when the animals were handled extensively and transported) until Day 14 (Franzman, 1970). The heart rate of newly-captured European starlings was high compared to birds held for more than a year in captivity but decreased to the level of long-term captives within 24 hours (Dickens and Romero, 2009). The adrenomedullary response to captivity was slightly different in house sparrows. Daytime heart rate was elevated above 1 month captive levels for at least 7 days post-capture (Fischer and Romero, 2016). These data led to a long-term repeated-measures investigation during the first 6 weeks of captivity (Fischer *et al*., 2018). Heart rate tended to decrease until Day 18, then plateaued. Furthermore, there was a more profound effect on the heart rate response to a sudden noise in the starling study. While long-term captives showed a robust increase in heart rate after a loud noise, a typical adrenomedullary response, newly-captured birds had a virtually eliminated heart rate response for at least 10 days (Dickens and Romero, 2009). A reduction in the startle response (as demonstrated in European starlings) could have negative consequences for animals that are released from captivity into the wild (Dickens *et al*., 2009a). The adrenomedullary response to sudden noises or other startling events is an adaptation that allows animals to survive sudden traumatic events, such as predator attacks or conspecific aggression. An impaired startle response could result in death if it persists after the animals are released from captivity.

Overall, there are few studies examining the effects of captivity on the adrenomedullary response. The patterns we see in European starlings and house sparrows are different—it does not appear that there is a consistent heart rate response to captivity in passerine birds, much less in all vertebrates. We believe this is an area ripe for future studies. As telemetry equipment becomes cheaper and more available, we hope to see more investigations into the adrenomedullary response to captivity and other stressors.

### Effects of captivity on seasonality of hormone regulation

Some studies examined seasonal differences in the response to captivity. Table 6 shows that the time of year when animals are introduced to captivity can have a profound effect on hormonal changes. For example, baseline GCs might increase when free-living birds are in molt, decrease when free-living birds are breeding, and not change when free-living birds are captured during the winter or spring (Romero and Wingfield, 1999). Furthermore, Table 6 indicates that there is no consistent pattern across seasons or taxonomic groups. The implications of these differences are currently unknown, but the season of capture might partly explain the large variation across studies summarized in Figs 2–4. Understanding why there are seasonal differences in the acclimation to captivity would be an important contribution to this field.

**Table 6:** Seasonal effects of captivity

**Hormone**	**Species**		**How was free-living GCs established?**	**Captivity duration**	**Pre-breeding**	**Breeding**	**Post-breeding/molt**	**Winter**	**Citation**
Baseline GCs	Mammals	Canada lynx (*Lynx canadensis*) females	Free-living population	Long term (unknown)	−			↑	(Fanson *et al*., 2012)
		Canada lynx (*Lynx canadensis*) males	Free-living population	Long term (unknown)	↑			↑	(Fanson *et al*., 2012)
		Harbor seal (*Phoca vitulina*)	Free-living population	Long term (unknown)	↓	↓	↓		(Gardiner and Hall, 1997)
	Birds	White-crowned sparrow (*Zonotrichia leucophrys gambelii*)	Free-living population	4 months at start	−	↓	↑	−	(Romero and Wingfield, 1999)
		House sparrow (*Passer domesticus*)	Same individual pre-capture	5 days	↑	−	−	−	(Lattin *et al*., 2012)
	Reptiles	Duvaucel’s gecoks (*Hoplodactylus duvaucelli*)	Free-living population	>1 year at the start	−	↑		−	(Barry *et al*., 2010)
		Tuatara (*Sphenodon punctatus*) (♀)	Free-living population	Unknown		↑		−	(Tyrrell and Cree, 1994)
		Tuatara (*Sphenodon punctatus*) (♂)	Free-living population	Unknown		−		−	(Tyrrell and Cree, 1994)
	Amphibians	Eastern red-spotted newt (*Notophthalmus viridescens*)	Free-living population	2 months to > 1 year	↑	↑		−	(Berner *et al*., 2013)
		Green frog (*Rana esculenta*)	Same individual pre-capture	3 days	↑	↑	↑		(Zerani *et al*., 1991)
	Fish	Winter flounder (*Pseudopleuronectes americanus*) (juvenile)	Free-living population	2 months at start	↓^1^		−		(Plante *et al*., 2003)
Acute stress GCs	Birds	White-crowned sparrow (*Zonotrichia leucophrys gambelii*)	Free-living population	4 months at start	−	↓	↑	−	(Romero and Wingfield, 1999)
		House sparrow (*Passer domesticus*)	Same individual pre- capture	5 days	−	−	−	↓	(Lattin *et al*., 2012)
	Amphibians	Eastern red-spotted newt (*Notophthalmus viridescens*)	Free-living population	>1 year	↑	↑		−	(Berner *et al*., 2013)
	Fish	Winter flounder (*Pseudopleuronectes americanus*) (juvenile)	Free-living population	2 months at start	−		−		(Plante *et al*., 2003)
T	Mammals	Armadillos (*Dasypus novemcinctus*)	Free-living population	2 weeks–3 years	−	−	−	−	(Czekala *et al*., 1980)
	Amphibians	Green frog (*Rana esculenta*) (♀)	Same individual pre-capture	3 days	↓	−	−		(Zerani *et al*., 1991)
		Amphibian: green frog (*Rana esculenta*) (♂)	Same individual pre-capture	3 days	↓	↓	↓		(Zerani *et al*., 1991)
E2	Amphibians	Amphibian: green frog (*Rana esculenta*) (♀)	Same individual pre-capture	3 days	↓	↓	↓		(Zerani *et al*., 1991)
		Amphibian: green frog (*Rana esculenta*) (♂)	Same individual pre-capture	3 days	−	−	↓		(Zerani *et al*., 1991)

Arrows indicate direction of change in captive animals relative to an established free-living level. Captive and wild measurements were taken at the same time of year. GCs glucocorticoids; T testosterone; E2 estradiol.

^1^Delay in acquiring wild baseline.

### Other physiological consequences of captivity

Some studies, primarily in marine mammals, reported the effects of captivity on thyroid hormone. Unfortunately, there is not a consistent impact. For example, one study of beluga whales reported that thyroid hormone decreased over the first few days of captivity, but increased to a long-term stable level by day 11 (Orlov *et al*., 1991), whereas another study reported that thyroid hormone decreased within the first few days and remained low throughout 10 weeks of captivity (St Aubin and Geraci, 1988). Similarly, rehabilitated harbor seal juveniles held in captivity for 4 months had lower thyroid hormone than free-living juveniles (Trumble et al., 2013). In contrast, long-term captive harbor porpoises had the same thyroid hormone levels as wild populations (Siebert *et al*., 2011) and in female brushtail possums, thyroid hormone was elevated from Weeks 6–13, the same period when the animals were regaining weight they had lost in captivity (Baker *et al*., 1998). Clearly, more work is needed to determine the effect of captivity on thyroid hormone regulation.

Anatomical changes may also occur in captivity. Mountain chickadees showed remarkable reduction in hippocampal volume after 4 months of captivity (LaDage *et al*., 2009), an effect mimicked by black-capped chickadees after 4–6 weeks in captivity (Tarr *et al*., 2009). In neither species was the telencephalon affected—the effect was localized to the part of the brain involved in location-based memory tasks. This effect persisted even when the environment was enriched to include memory tasks (LaDage *et al*., 2009).

Captivity can lead to various pathologies. In a histological study of mouse lemurs that died spontaneously in captivity, lesions in the kidney were strongly correlated with captivity duration and with adrenal size (Perret, 1982). The investigator also concluded that cardiac disease may result from chronic adrenomedullary stimulation, although they did not measure hormone concentrations directly (Perret, 1982). Similarly, herring gulls developed amyloid deposits in the blood vessels of their spleens after 28 days in captivity (Hoffman and Leighton, 1985). There may be many more hidden anatomical changes resulting from captivity, but few studies have looked for them.

Finally, recent data indicates that captivity can have profound effects at the DNA level. Bringing house sparrows into captivity resulted in an approximately doubling of DNA damage in red blood cells (Gormally *et al*., 2019). The impact of this damage on the individual remains to be determined.

### Amelioration of captivity stress

Captivity can cause a wide array of physiological changes in wild animals that are consistent with chronic stress and are likely to be detrimental to health. However, can anything be done to prevent these changes? Is there a way to protect animals from the negative consequences of captivity stress? While this is not an exhaustive review of the solutions that have been tried, we offer some ideas that have been attempted to relieve symptoms of chronic stress due to captivity conditions.

Adjusting the physical conditions of captivity may be one of the simplest ways to reduce symptoms of chronic stress. Transferal from outdoors cages to indoors cages led to reduced reproductive hormones and behaviors in long term captive European starlings (Dickens and Bentley, 2014) and to weight loss and reduced immune function in water voles (Moorhouse et al., 2007). Cage size and density are also important for the development of chronic stress. High density housing during the initial captivity period resulted in elevated GCs compared to low density housing in flounders (Nester Bolasina, 2011) and wedge sole (Herrera *et al*., 2016). However, reducing density by caging animals individually can have negative consequences, particularly in social species. Housing brushtail possums in groups eliminated the infection, weight loss and mortality that were seen when the animals were caged individually (*McLeod et al*., 1997). In male brown headed cowbirds, adding a female to the cage (previously solo housed) resulted in reduced plasma GCs, as well as increased testicular regrowth in photostimulated males (Dufty Jr and Wingfield, 1986).

Many animals benefit from the use of behavioral enrichments to reduce abnormal behaviors that develop in captivity (reviewed in Mason *et al*., 2007). Enrichments have become standard practice in zoo environments and situations where animals are held long-term or bred in captivity. Enrichments consist of providing animals with the means and motivation to practice a full range of natural behaviors, such as foraging opportunities, exercise opportunities and places to bathe or dust bathe. Even in temporary or laboratory conditions, environmental enrichments can be relatively easy to supply. However, we were unable to find any papers where the physiological benefits of enrichment techniques were specifically tested in newly captured animals. Using these techniques to accelerate the adjustment to captivity would be an exciting avenue for future research.

Lighting conditions may be very important for visual species. European starlings show more behavioral signs of chronic stress under fluorescent lights with a low flicker rate than a high flicker rate (Evans *et al*., 2012), but the low flicker rate does not elicit a GC response (Greenwood *et al*., 2004). Ultraviolet-deficient lighting resulted in higher baseline GCs in European starlings, although immediately after capture, this stressor may be too subtle to make a difference compared with the other stressors of captivity (Maddocks *et al*., 2002). Temperature conditions should also be carefully considered, particularly for poikilotherms. Warm conditions during the initial transfer to captivity resulted in high mortality in sardines (Marcalo *et al*., 2008) and higher GCs in cane toads (Narayan *et al*., 2012).

Overall, by matching captivity conditions as closely as possible to conditions in the wild, with roomy cages, exposure to naturalistic lighting and temperature conditions and animal densities kept relatively low, many animals will be better able to adjust to captivity and may have reduced chronic stress as a result. However, naturalistic housing conditions may be impractical for many situations. Furthermore, some stressors associated with captivity may be unavoidable. For example, nearly any visual or auditory contact with handlers resulted in a heart rate increase in two red-shouldered hawks (Patton *et al*., 1985). Therefore, in some cases, it might be beneficial to use pharmaceuticals to reduce symptoms of chronic stress.

Tranquilizers or sedatives are perhaps the most obvious drug classes to consider using in newly-captured animals. However, these may not be particularly effective at eliminating chronic stress symptoms. A long-acting neuroleptic did not result in many physiological changes in newly-caught otters (Fernandez-Moran *et al*., 2004). Tranquilizers did not impact any physiological variable in newly caught impala (Knox *et al*., 1990) or red-necked wallabies (Holz and Barnett, 1996), although they reduced behavioral agitation to human approach and handling in the later study. Similarly, a long-acting tranquilizer changed behavior but not heart rate response to human approach in captive wildebeest (Laubscher *et al*., 2016). The anxiolytic and sedative diazepam did not affect GCs, heart rate, heart rate variability or activity in house sparrows during the first week of captivity (unpublished personal data). Overall, tranquilizers and sedatives do not appear to have long-term physiological benefits in captive animals. However, they may be useful in the short term. For example, by reducing physical agitation, they may prevent animals from injuring themselves during transport (e.g. in nurse sharks being moved into captivity; Smith, 1992) or during necessary handling by humans (e.g. in red-necked wallabies; Holz and Barnett, 1996).

Another strategy for pharmaceutical reduction of symptoms of chronic stress may be to chemically block the hormones of the stress response. The chemical agent mitotane causes a reversible chemical adrenalectomy, which drastically reduces circulating GCs (Sanderson, 2006). In house sparrows treated with mitotane immediately upon capture, baseline and stress induced GCs were drastically reduced during the initial captivity period, but recovered to the level of untreated birds by Day 10 of captivity (Breuner *et al*., 2000). We investigated the effects of mitotane treatment during the first 7 days of captivity in house sparrows and found that it reduced resting heart rate even when it did not cause the expected dramatic decrease in GC (unpublished personal data). The adrenomedullary response can also be pharmaceutically reduced by blocking the receptors of epinephrine and norepinephrine. We used alpha- and beta-blockers (which interfere with binding of epinephrine and norepinephrine to their receptors) during the first week to block chronic captivity stress in house sparrows. We found that while the beta-blocker propranolol had no effect on heart rate, it did prevent the increase in baseline GCs that we typically see in newly-captured members of this species (Fischer and Romero, 2016).

### The persistence of captivity effects after release

The physiological changes caused by captivity can persist even after animals have been released back into the wild. Chukar partridges that were held in captivity 10 days and then released to a new location than where they had originated had lasting changes to their GC regulation (decreased negative feedback for at least 30 days, Dickens *et al*., 2009a). Red foxes that were kept in captivity for 2 to 8 weeks were less likely to establish a stable territory upon release than foxes that were caught and immediately released (Tolhurst *et al*., 2016). River otters kept in captivity for 10 months had lower survival than otters not kept in captivity (Ben-David *et al*., 2002). The captivity effect was strong enough that crude oil ingestion (mimicking the state of oiled otters in rehabilitation) had no further effect on survival (Ben-David *et al*., 2002). Rehabilitated barn owls (Fajardo *et al*., 2000) and guillemots (Wernham *et al*., 1997) had much shorter life expectancies than wild birds.

However, captivity may not necessarily have lasting negative impacts. In Grevy’s zebra, fecal GC metabolites were elevated in captivity, but decreased back to the wild norm quickly after release (Franceschini *et al*., 2008). Similarly, released Eastern Bettongs decreased GC metabolites after release from a period of over 30 days of captivity (Batson *et al*., 2017). Hermann’s tortoises kept in captivity for 2–8 years following an injury showed no difference in movement, thermoregulation or body condition compared to free-living animals after release to the wild (Lepeigneul *et al*., 2014). Captivity up to 3 months did not affect survival in Stellar’s sea lions (Shuert *et al*., 2015). Captivity may even have positive effects in some cases. For example, hedgehogs were more likely to survive a translocation event if they were held in captivity for greater than 1 month compared to those held <6 days (Molony *et al*., 2006).

Whether an animal will be permanently negatively impacted by captivity or not may depend on the captivity conditions, species, time of year, method of release or individual effects. Wild rabbits held for 2, 4, 6 or 8 weeks in quarantine before release did not differ in survival probability (Calvete *et al*., 2005). In another study in that species, GCs did not change over the course of a quarantine period, but animals with higher plasma and fecal GCs were more likely to survive, even though they had worse body condition (Cabezas *et al*., 2007). Saddlebacks were more likely to survive post-release when they had a robust GC response to a standardized acute stressor (Adams *et al*., 2010). Therefore, captivity may have more profound effects on survival if it negatively and permanently changes GC regulation.

## Conclusions

Captivity can cause weight loss, persistent changes in baseline and integrated GCs, changes in the immune system and reproductive suppression. These effects can last for months or years in some species, indicating that some species may never truly adjust to captivity conditions. The welfare implications of chronic captivity stress are obvious, and zoos and other institutions that hold animals in captivity long-term generally have strategies in place to minimize captivity stress. Breeding facilities (for conservation, research and agriculture/fisheries) are particularly invested in reducing chronic captivity stress, given its profound impact on the reproductive system. Figure 3 indicates that many species may continue to have elevated GCs months or years after capture, while Figs 2 and 4 suggest that most animals will recover from the weight loss and elevated N or H:L ratios caused by the initial transfer to captivity. Given that weight loss and changes to N or H:L ratio are affected by GCs, it is possible that with continuing high GC concentrations, sensitivity to these hormones decreases in captive animals. The reproductive system tends to be negatively impacted by captivity, presumably because of elevated GC hormones. The negative effects of captivity are species-specific, some species adjust to captivity while others do not (see also Mason, 2010).

A captive animal may be physiologically quite different than a wild animal (Calisi and Bentley, 2009). Therefore, the confounding effects of captivity must be considered in physiological studies using captive wild animals, even when stress is not the focus of research. Animals that are held in captivity for research might respond quite differently to a range of experimental treatments than a wild, free-living individual would. For example, environmental contaminants had different effects on wild and captive sea otters (Levin *et al*., 2007), and experimentally induced chronic stress caused a change in fecal GCs in free-living but not captive European starlings (Cyr and Romero, 2008).

The existing literature indicates that the effects of captivity on physiology are inconsistent. Some of the differences between animals that adjust and do not adjust to captivity might be explained by life-history features of the different species (see Mason, 2010). For example, captive predators that have large ranges in nature tend to show more behavioral anomalies and more infant mortality than those that naturally have smaller ranges (Clubb and Mason, 2003). However, it may be possible to improve the physiological outcome for newly-captured animals by adjusting the season of capture, improving and enriching housing, allowing for an appropriate adjustment period, and possibly by the careful use of pharmaceuticals. Captivity stress will continue to be a factor in captive animal research, and the conditions, timing and duration of captivity must be considered as experiments are designed and interpreted.

Unfortunately, the results of this literature review do not suggest useful overall and/or generalized guidelines to wildlife managers. The overall picture is that wild animals acclimate to captivity in a highly species-specific manner. However, the most important conclusion from this review is that collecting multiple measures of physiology, rather than restricting studies to a single measure (e.g. GC concentrations), will provide a better picture of how well an individual or species is, or is not, coping with introduction to captivity.

## Funding

This work was supported by the U.S. National Science Foundation [grant number IOS-1655269 to L.M.R.].
